# Effect of Water Temperature and Time during Heating on Mass Loss and Rheology of Cheese Curds

**DOI:** 10.3390/foods10112881

**Published:** 2021-11-22

**Authors:** Ran Feng, Søren K. Lillevang, Lilia Ahrné

**Affiliations:** 1 Department of Food Science, University of Copenhagen, Rolighedsvej 26, 1958 Frederiksberg, Denmark; ran.feng@food.ku.dk; 2 Arla Foods Amba, Argo Food Park 19, 8200 Aarhus N, Denmark; sklv@arlafoods.com

**Keywords:** cheese curd, cooking time and temperature, mass loss, rheology

## Abstract

During the manufacturing of mozzarella, cheese curds are heated to the desired stretching temperature traditionally by immersion in water, which influences the curd characteristics before stretching, and consequently the final cheese properties. In this study, cheese curds were immersed in hot water at 60, 70, 80 and 90 °C up to 16 min and the kinetics of mass loss and changes of rheological properties were investigated. The total mass of cooked curds increased up to 10% during the first minute, independent of the temperature, as a consequence of water retention. Fat was the main component lost into the cooking water (<3.5% *w/w*), while the concentration of protein increased up to 3.4% (*w/w*) compared to uncooked curds due to the loss of other components. Curds macrostructure during cooking showed that curds fully fuse at 70 °C/4 min; 80 °C/2 min and 90 °C/1 min, while after intensive cooking (>8 min) they lost the ability to fuse as a consequence of protein contraction and fat loss. Storage modulus, representing the curd strength, was dependent on cooking temperature and positively, and linearly, correlated with curd protein content (21.7–24.9%). This work shows the potential to modify curd composition and structure, which will have consequences for further processing and final product properties.

## 1. Introduction

Pasta-filata cheese processing involves a combined cooking-stretching step, during which the cheese curd is first cooked in hot water followed by shear stretching. The purpose of the cooking is to achieve the gel-sol transition temperature (i.e., the temperature where storage modulus G′ equals loss modulus, G′′) that is necessary to plasticize the cheese curd during stretching [1]. Typically, curd cooking is done in hot water, with a curd-to-water ratio of 1 to 1.4 [2,3,4], at temperatures that usually range from 60 °C to 85 °C and time ranging from 4 to 27 min [5,6]. Phase transition, molecular redistribution and compositional loss occur in cheese curd during this process: the curd mass transits from a viscoelastic solid to a viscoelastic liquid state during heating due to weakening of protein phase [7]; fat coalescence arises with increasing curd temperatures [8]; protein, fat, and calcium migrate from the curd into the water [6]. The extent of these transformations and mass loss depend on the equipment and processing parameters selected that determine the heat and mass transfer from the heating medium to the curd. 

Combined thermal and mechanical effects on cheese composition have been reported in literature since cooking-stretching is usually applied in a single unit operation in the dairy industry. Bähler et al. (2013) reported that an increase in cooking temperature from 55 to 70 °C reduced the cheese yield from 0.88 to 0.59 g/g [4]. Banville et al. (2016) reported higher protein and lower fat contents of the cheeses after thermomechanical processing in 60–70 °C water, and an average weight loss of 10% was observed that was mainly caused by the fat loss [9]. Our previous study showed that increasing cooking residence time of cheese curd in cooker-stretcher induced increased water and decreased protein and fat contents of the cheese, by respectively 5.6%, 5.4% and 8.8% (*w/w*) [6]. However, due to the various equipment and processing parameters used, as well as the combination of thermal and mechanical effects, it is difficult to extract the effects of the cooking process alone on mass loss and cheese curd composition. 

There is also limited information about the rheological properties of the curd during the cooking process, although the rheological properties of cheese have been extensively studied. Cheese is a viscoelastic material and its rheological properties have been considered more crucial than flavor attributes for consumer preferences [7]. The number, strength, and type of bonds between casein molecules constitute the basis of the cheese rheological properties [7], while these attributes are influenced by cheese composition and microstructure. Influence of fat on cheese rigidity (storage modulus, G′) was found to be dependent on temperature: increased fat content induced increased G′ at 10 °C, whereas the effect was not apparent at 25 °C because G′ of fat approaches that of the protein gel [10]. Gel-sol transition temperature was found to increase with calcium-to-protein ratio [11], and the higher transition temperature indicates more difficult plasticization and melting due to higher proportion of colloidal Ca and strengthened protein phase [7,12]. Hence, changes of rheological properties of the curd during the cooking process are important to investigate as they will determine the structural arrangements of the curd at the starting point of further processing. 

Although, the cooking step determines the curd yield, composition, structural properties and consequently influences the functionality of the final cheese product, knowledge about the mass loss during the cooking process at different temperatures, as well as consequences for physical–chemical and structural properties of the curd have not been assessed. This knowledge is relevant to understand the performance of curds during further processing (e.g., stretching) and develop innovative dairy products. Thus, the current study aims to determine the kinetics of mass loss from the curd during cooking at temperatures from 60 to 90 °C up to 16 min and consequences for structural and rheological properties of the curd.

## 2. Materials and Methods

### 2.1. Sample Preparation and Cooking Process

Frozen (−20 °C) ‘Cagliata’ mozzarella curd (24.0% protein, 27.0% fat, 43.7% water, 0.82% Ca, 0.69% NaCl) was provided by Arla Foods (Fredericia, Denmark). The curd was fermented by starter culture brined in NaCl and had a pH of 5.6. It was stored in a 4 °C refrigerator for three days before processing in order to fully defrost. The curd was then cut into 1 cm^3^ cubes using a wire cutter and 30 g of curd was cooked with 2.5% (*w/v*) NaCl solution at 60, 70, 80 and 90 °C in a shaking water bath (Grant Instruments, Cambridge, UK) at 125 rpm. The curd-to-water ratio was 1:1 (*w/w*) (30 g curd and 30 g water) and each experiment was performed in duplicate. Based on pre-experiments, the liquid was separated from the curd using a sieve after 1-, 2-, 4-, 8-, 12- and 16-min cooking time, and stored at −20 °C until composition analysis. The cooked curd was weighted, photographed, transferred into a cylindrical cup and stored at 4 °C overnight for rheological measurements on the following day.

### 2.2. Composition Analysis of Cooking Water

Nitrogen, fat and water content were determined by the Kjeldahl [13], Gerber [14] and the oven-drying [15] methods, respectively. Protein was quantified by Kjeldahl using a nitrogen conversion factor of 6.38. The water content was determined at 105 °C. Calcium and sodium content was measured by Inductively Coupled Plasma Optical Emission Spectroscopy (ICP-OES; Agilent Technologies, Santa Clara, CA, USA). Before analysis, 1 g of sample was digested with 8 ml HNO_3_ (65%) and 2 ml HCl (37%) using a Multiwave GO microwave (Anton Paar, Graz, Austria). Standards in a range of 0.04–20 mg L^−1^ were prepared from multi-element (Ag, Al, B, Ba, Bi, Ca, Cd, Co, Cr, Cu, Fe, Ga, In, K, Li, Mg, Mn, Na, Ni, Pb, Sr, Tl, Zn) standard solution IV from Merck KGaA. Standard curves were determined at the wavelengths of 396.847 nm and 588.995 nm for calcium and sodium, respectively. All analysis were performed in duplicate.

### 2.3. Rheological Properties of Cooked Curd

Rheological properties were determined by the methods of Feng et al. (2021) with some modifications [6]. The rheological properties of the cheeses were studied on a rheometer (Discovery HR-2, TA Instruments, New Castle, DE, USA) with 25 mm diameter serrated parallel plates. Disc-shaped cheese samples of 25 mm diameter and ~2 mm thickness were prepared and equilibrated for 5 min at the test temperature. A 1N normal force was used to define the measurement gap. A ring of paraffin oil was placed around the sample periphery to avoid moisture loss during rheological measurements.

Frequency sweeps were conducted by applying frequencies in descending order from 50 Hz to 0.1 Hz at 20 °C using 0.1% strain amplitude (within linear viscoelastic region). The data of storage modulus G′ was fitted to the following equation:

G′ = G′_1_ · ω^n^ (1)

where *G*′_1_ is the storage modulus measured at 1 Hz, ω is the frequency in Hz, and *n* is the degree of frequency dependence.

In temperature sweeps, strain and frequency were 0.1% and 1 Hz respectively and the temperature was increased from 20 to 100 °C. The rate of the Peltier heating system was set at 3 °C·min^−1^. All rheological measurements were conducted at least in duplicate.

### 2.4. Thermal Analysis of Uncooked Curd by Differential Scanning Calorimetry

The thermal properties of the uncooked Cagliata curd were evaluated by differential scanning calorimetry (DSC 1, Star^e^ System, Mettler Toledo, Glostrup, Denmark). Samples of 10–15 mg were placed in aluminum pans and heated from 20 °C up to 90 °C with a heating rate of 5 °C·min*^−^*^1^. An empty sealed aluminum pan was used as reference in every test. Heat flow (W·g^−1^) versus temperature curves was obtained. Triplicate was measured for the curd sample.

### 2.5. Statistical Analysis

One-way analysis of variance (ANOVA) followed by a Duncan test was done to verify differences between means using IBM SPSS Statistics 28 (IBM Corporation, Somers, NY, USA). Differences were considered significant at the probability level *p* < 0.05.

## 3. Results and Discussion

### 3.1. Mass Transfer from Curd to the Cooking Water

The total mass loss (yield) (Table 1) shows an initial mass gain, independent of the cooking water temperature during the first 4 or 8 min of cooking up to 10% followed by a mass loss up to 7% for curds cooked for 16 min at 70 or 80 °C. These results can be explained by the hydration of the curds when immersed in water and by the changes in the individual components of the curd as will be discussed below.

**Figure 1:**
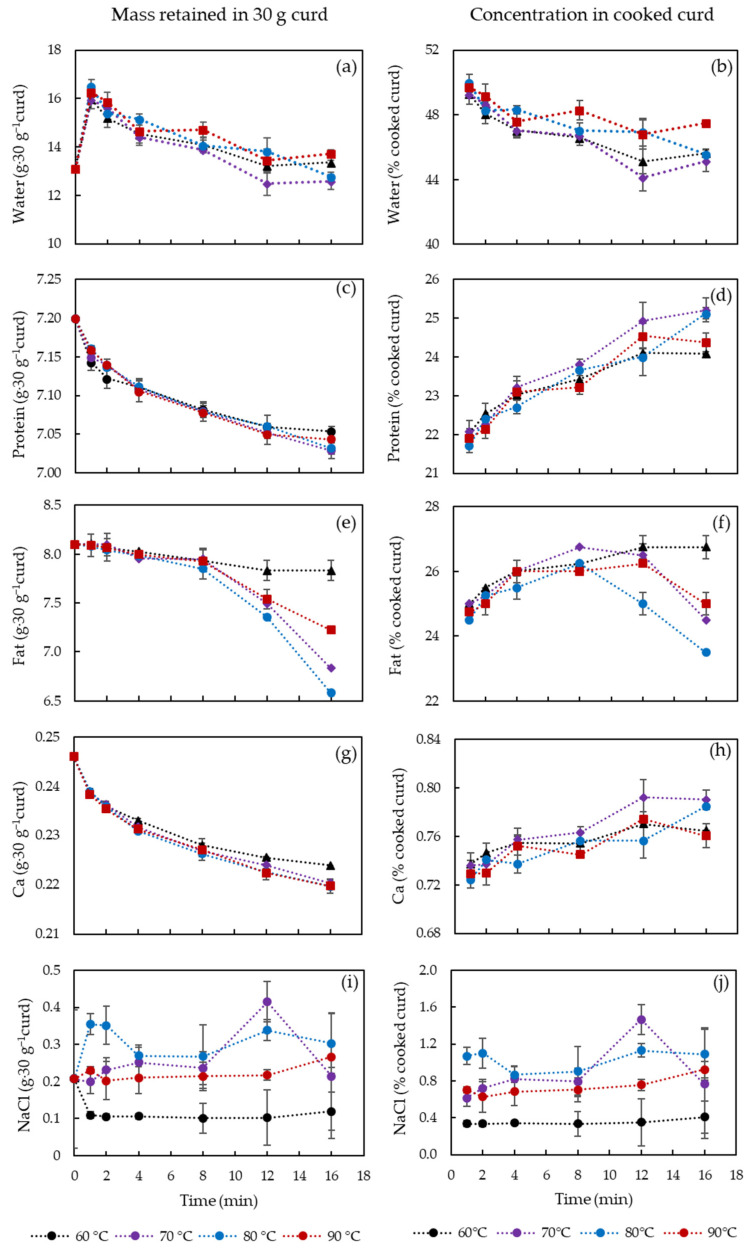
Content (%, *w/w*) of water, protein, fat, calcium and NaCl retained in curd at varied cooking conditions. (**a**,**c**,**e**,**g**,**i**) show the content in 30 g original curd while (**b**,**d**,**f**,**h**,**j**) show the % concentration in final curd. The error bars indicate standard deviations.

Water, protein, fat, Ca in the curds after cooking at 60, 70, 80 and 90 °C for 1, 2, 4, 8, 12 and 16 min are shown in Figure 1. The figures on the left side (Figure 1a,c,e,g,i) show the mass in grams of each component remained in the 30 g original curd, while those on the right side (Figure 1b,d,f,h,j) show the final % (*w/w*) of each component in the cooked curd.

**Figure 2:**
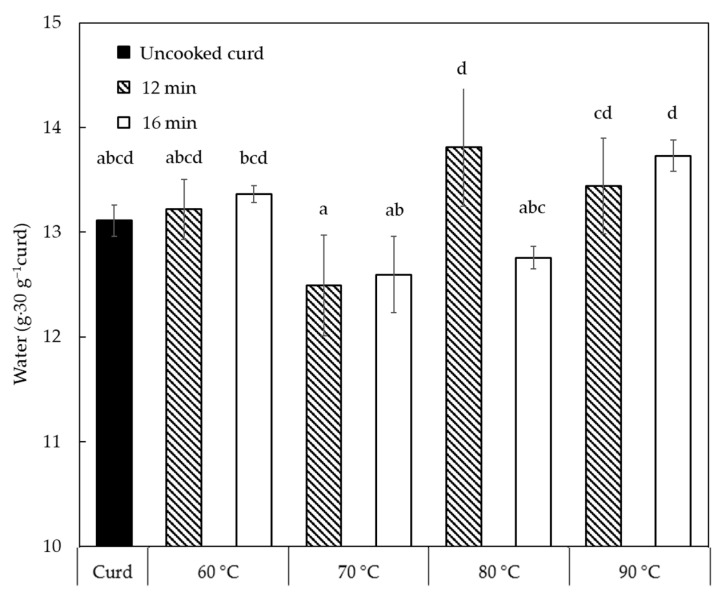
Water content retained in 30 g curd at varied cooking temperatures for 12–16 min. Values with different superscripts differ significantly (*p* < 0.05). The error bars indicate standard deviations.

A significant increase of water content from 13.1 g to 16.2–16.5 g (*p* < 0.05) in 30 g of curd is observed in the first minute of cooking (Figure 1a, because cooking water gets attached to the curd surface and entrapped between the individual curd cubes. With the longer cooking until 8 min, water content in the cooked curds was always above the water content level of the uncooked curd due to the presence of surface and entrapped cooking water. The amount of the entrapped water decreased from 16.2–16.5 g (1 min) to 12.6–13.7 g (16 min) in 30 g curd with increased cooking time (*p* < 0.05) since the cooked curd gradually lost the ability to retain the water. The cooking temperature did not play a significant role in affecting the water content until 12 min, likely because of the surface temperature. To identify the temperature effect after 12 min, Figure 2 shows the water content in 30 g curds cooked for 12–16 min in comparison with uncooked curds. No significant difference between the cooked and uncooked curds was observed, due to the large standard deviations, though the curds processed at 70 °C/12–16 min displayed significantly lower (*p* < 0.05) water content (12.5–12.6 g) compared to curds obtained at 80 °C/12 min and 90 °C/12–16 min (13.4–13.8 g). As previously reported, cheese heating at 60–100 °C can induce contraction and shrinkage of the protein network and simultaneous expulsion of water as a result of increased hydrophobic interactions between casein molecules [16]. Hence, the slightly lower water content at 70 °C might be attributed to the structural changes of the casein network that expelled water. However, curds cooked at 80–90 °C presented a higher water content probably because of the sudden shrinkage of the curd protein matrix and skin formation that hindered syneresis at such high temperatures [17,18]. These changes in the casein network might be a result of Ca precipitation due to heat treatment that affected Ca-protein interactions [19]. This is also visible in Figure 1b that shows that the water in the cooked curd reduced by 5% during cooking at 60–80 °C, whereas to a less extent (2%) at 90 °C due to the formed skin as mentioned.

The protein content of the curd was mainly affected by the cooking time and not by the temperature in the range studied (Figure 1c). The rate of protein release into the water slowed down after 2 min cooking and tends to stabilize for longer cooking times, suggesting that most protein losses occurred at the curd surface at initial cooking phase. The absolute protein loss in 30 g curd was only 0.17 g, which represent a 3.5% increase of protein content (Figure 1d) in the cooked curd as a result of loss of other components, i.e., fat, etc. The cooking temperature had little effect on protein content, only at 16 min cooking statistically significant differences were observed between 60/90 °C and 70/80 °C (*p* < 0.05). This could be an indication of a weakened protein matrix that requires further studies in the future.

Fat was the main component lost into the cooking water, which at maximum decreased from 8.1 g to 6.6 g in 30 g curd for 80 °C as cooking time increased up to 16 min (Figure 1e). Longer cooking resulted in increased curd temperature and thus enhanced fat mobility. The highly mobile fat can, therefore, lead to fat coalescence and free oil release [4]. Different from protein content, fat content exhibited a sudden decrease at 8 min cooking for all temperatures, except 60 °C, suggesting that the coalesced fat is released from the curd casein matrix. Similar to water and protein kinetics, fat content at 90 °C decreased to a less extent compared to that at 70 and 80 °C, which may be due to the fact that fat migration is limited by the toughened protein matrix and skin formation at surface of the curds [20]. Thus, at cooking temperature of 90 °C, the curd surface might contract, forming a skin that hindered the rate of fat release. Figure 1f shows that, in fact, the % of fat content in the cooked curds increased with cooking time up to 8 min and this was not strongly influenced by temperature, whereas after 8 min significant differences are observed. At 60 °C the % fat content is higher for curds cooked at 16 min compared to 1 min cooking, whereas at 70–90 °C a significant decrease of fat content is observed (*p* < 0.05). The increase in the initial times of cooking was simply due to the loss of other components, specially entrapped water, and relatively limited fat release. After 8–12 min, as all the curd mass reached the water temperature, significant fat loss occurred as observed in Figure 1e (*p* < 0.05).

**Figure 3:**
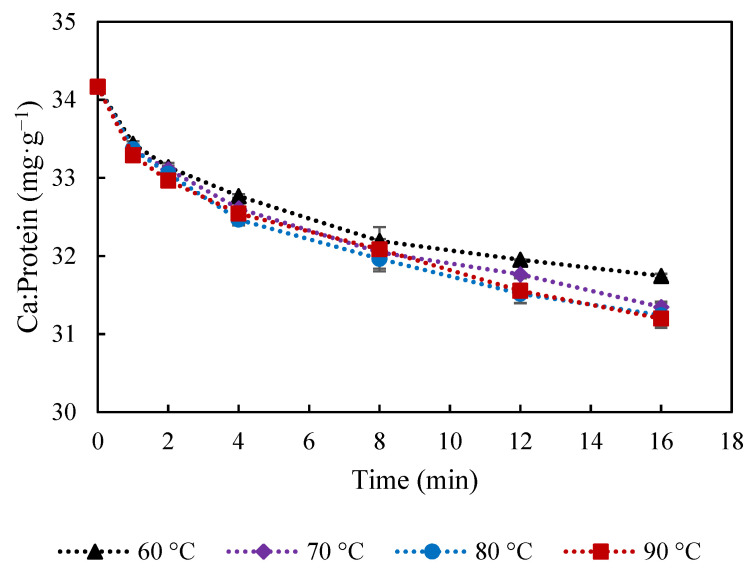
Calcium-to-protein ratio in mg calcium·g^−1^ protein at during cooking in water at 60, 70, 80 and 90 °C. The error bars indicate standard deviations.

The amount of Ca in the curd (Figure 1g) not only decreased with cooking time but also reached a lower value at temperatures of 70–90 °C, compared to 60 °C. Ca seems to have been continuously lost into the water together with protein, as similar trend is observed for Ca and protein (Figure 1d). Ca:protein ratio in the curd (Figure 3) was relatively less affected by the cooking temperature. It was reported that an increase from 15.8 to 31 mg Ca g^−1^ protein induced decreased gel-sol temperature from 60 to 51.7 °C [11]. Thus, the slight reduction of Ca:protein from 34.2 to 31.2 mg Ca g^−1^ protein is not expected to induce significant changes on the curd properties. However, the proportion of soluble and insoluble Ca might be different, which is highly affected by water and temperature, and has been reported to play an important role in melting properties of cheese [21].

Limited uptake or loss of NaCl from the curds to the water was observed during the cooking process, however, differences in salt content is observed at different temperatures (Figure 1i,j). After 16 min the cooked curds at 60 °C exhibited a lower NaCl content (0.4 ± 0.2% *w/w*) compared to cooked curds at 70, 80 and 90 °C curds that had respectively 0.8 ± 0.6%, 1.3 ± 0.3% and 0.9 ± 0.1%), which was also similar to the content after 1 min cooking. These differences are due, as previously discussed, to the surface water and entrapped cooking water that contained 2.5% NaCl. Therefore, at water temperature >70 °C acceleration of salting was observed.

### 3.2. Macrostructural and Rheological Properties of Curds during Water Cooking

**Figure 4:**
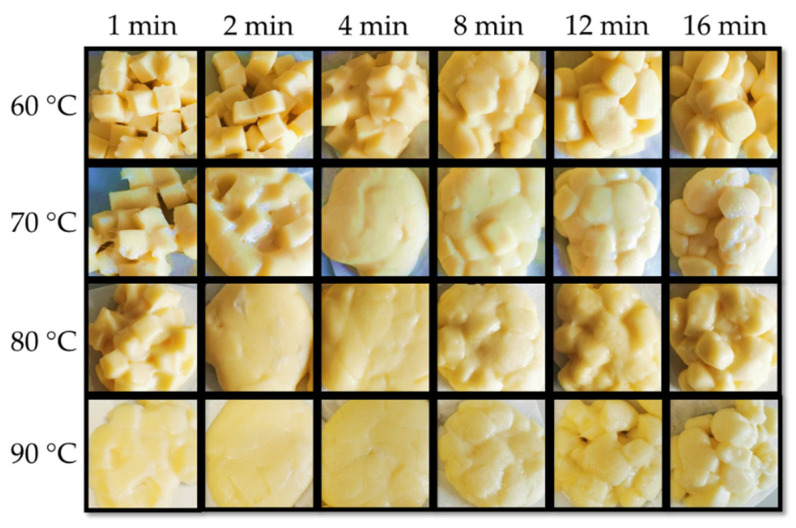
Appearance of curd after cooking at varied conditions.

The appearance of cooked curd after cooking at 60 to 90 °C/1–16 min is shown in Figure 4, and a summary of the time, at which the macrostructure transitions occurred are presented in Table 2. It was noticed that the curds were always kept separated in the cooking water during cooking. Independent of the temperature, a general observation is that, as cooking time proceeds, curd cubes were able to fuse into one block after being removed from the water. However, after a given cooking time, the curds lost the ability to fuse together and form a block, instead separated cube particles can be observed. At 60 °C, the curd kept the cube shape during the first 2 min, starting to melt together after 4 min. However, the curd at 60 °C did not fully fuse to the same extent as observed at other temperatures (70 °C/4 min and 80–90 °C/2 min). After 12 min cooking, the curd appeared as separated cube particles again with visible edges and the same occurred at earlier times for higher cooking temperatures, i.e., 8 min at 70–90 °C as displayed in Table 2. Furthermore, curds cooked at 90 °C for longer times (12–16 min) presented visually the coarsest particles with a rough surface, indicating extensive mass loss.

**Figure 5:**
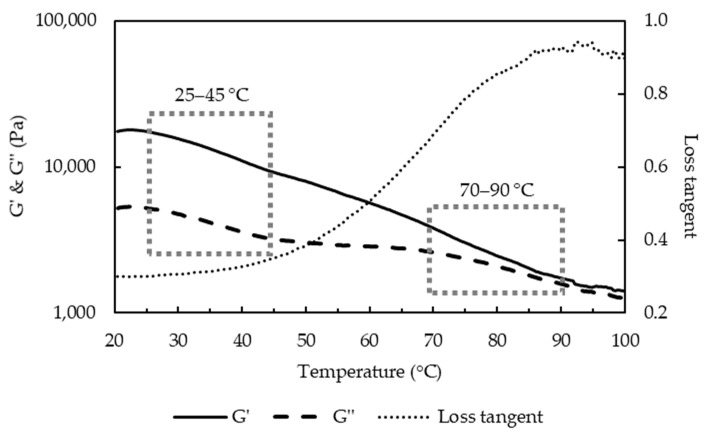
Storage and loss moduli during temperature sweep test on uncooked curd.

**Figure 6:**
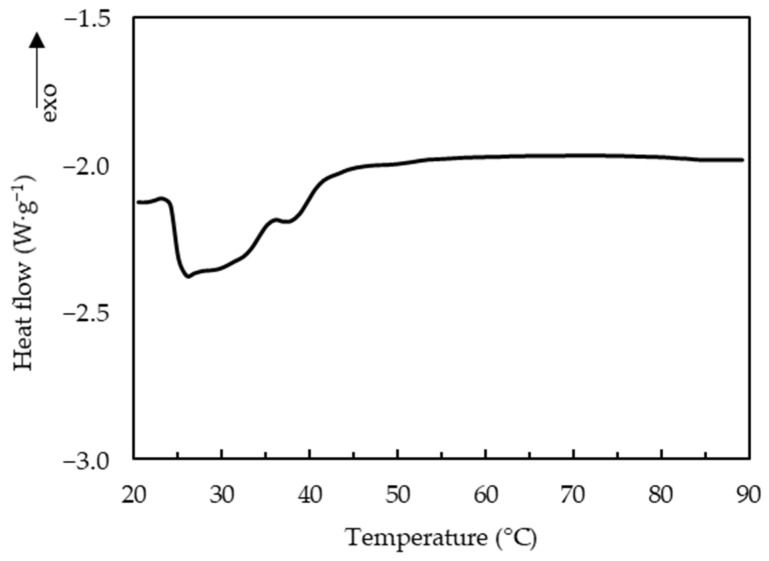
Differential scanning calorimetry curve of uncooked curd.

The melting of curds has been attributed to liquefaction of fat, and softening of protein matrix due to decreased number and strength of protein-protein bonds [7]. The thermo-rheological measurements of the uncooked curd (Figure 5) showed that G′ and G′′ decreased with the increase of temperature and loss tangent continuously increased during heating, reaching its maximum value (<1) at about 90 °C. A previous study of cheese has shown similar thermo-rheological behavior and two temperature ranges, one at 20–35 °C and another at 50–65 °C with a faster rate of decrease for moduli that were respectively attributed to melting of the fat phase and softening of the protein matrix [22]. The uncooked curd also showed two temperature ranges with faster decrease of moduli: the melting temperature of fat at 25–45 °C and the softening temperature of the protein matrix at 70–90 °C. DSC measurements of the uncooked curd confirmed that the fat melts (endothermic peaks) at 25.2–41.8 °C with a representative curve shown in Figure 6. 

Different from the cheese, uncooked curd contains tiny native fat globules that are protected by protein layers [6], which may justify the higher melting temperature. In addition, the higher Ca content (0.82%) and Ca:protein (34.2 mg Ca g^−1^ protein) in the uncooked curd suggested more protein bound to Ca together with an increased insoluble Ca content, which may also justify the higher protein softening temperature. Loss tangent was lower than the reported value of 2.64 for cheese [22]. The maximum loss tangent value is often regarded as an indicator of melt functionality. However, for the uncooked curd not stretched, a lower maximum value indicates a viscoelastic solid that was not plasticized. Fully melted curd was not observed at 60 °C (Figure 4), but only for temperature of 70 °C or above, which was consistent with the observed 70–90 °C protein softening range (Figure 5). The separation stage could be linked to the contraction of the casein matrix as mentioned in Section 3.1, and the formed skin hindered further fusion of protein matrix with visually coarser curd surface. Furthermore, the markedly reduced fat content at 8 min cooking (Figure 1e,f) likely contributed to this separation phenomenon. 

**Figure 7:**
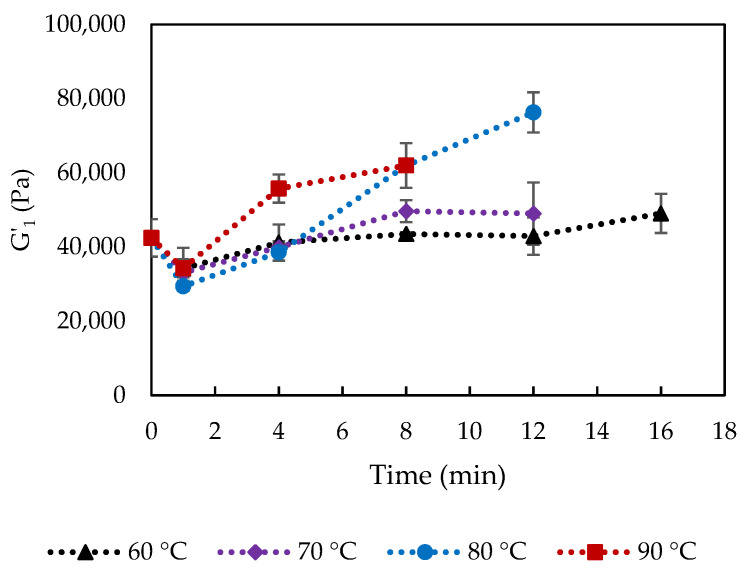
Storage modulus at 1 Hz (G′_1_) of curd cooked at varied conditions. The error bars indicate standard deviations.

The rheological properties of the cooked and uncooked curds were evaluated and Figure 7 shows the storage modulus at 1 Hz, G′_1_, of the cooked curd produced at 60, 70, 80 and 90 °C up to 16 min. Storage modulus reflects the total number and strength of protein-protein bonds in the protein matrix [7]. Curds cooked at 70 °C and 80 °C for 16 min and 90 °C for 12–16 min could not be measured because it was impossible to form a disk to be placed between the parallel plates of the rheometer. The reason could be the changes in calcium phosphate, inducing Ca-protein interactions, which caused a strongly contracted protein network with coarse curd surface that could not fuse to form a whole curd block anymore. Moreover, these conditions corresponded to extensive fat loss as previously discussed.

**Figure 8:**
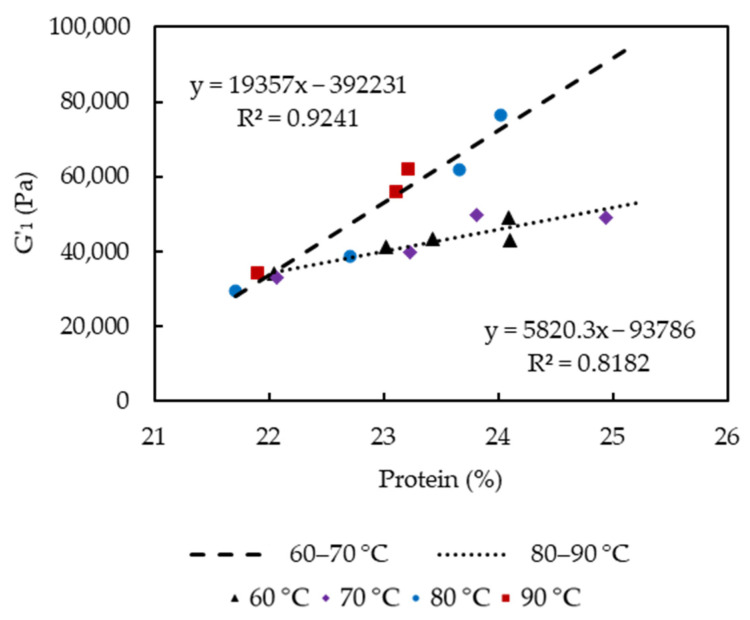
Plot of storage modulus versus curd protein content.

G′_1_ significantly increased as cooking time and temperature increased, which were related to the loss of entrapped water and fat, and relatively increased protein concentration, as well as the proportion of insoluble Ca. Lower moisture content was usually linked to increased cheese hardness and G′ due to a lower degree of casein hydration [23]. The observation was also in agreement with previous studies, which reported higher firmness of low-fat cheeses than that of full- or reduced-fat cheeses that indicated increases in the protein concentration and a more cross-linked structure [24,25]. Rheological properties of cheese at high temperatures are also associated with levels of insoluble Ca. The strength of Ca-protein binding was found to increase with increasing temperature [26], and G′ at 70 °C increased significantly with increasing concentrations of colloidal calcium phosphate [27]. The positive correlation between G′_1_ and protein content (21.7 to 24.9% *w/w*, Figure 8) in the cooked curd is temperature dependent, showing that enhanced protein-protein interactions as protein content increased have contributed to the increased strength of the protein network. A rapid increase of G′_1_ is observed at higher cooking temperatures 80–90 °C in comparison to 60–70 °C, as shown by the two trend lines for curds cooked at 60–70 °C and 80–90 °C instead of one for all the curds. The higher slope for 80–90 °C treated curds could be caused by the precipitation of the soluble Ca that led to Ca/phosphate-mediated protein-protein interactions between casein molecules [16].

**Figure 9:**
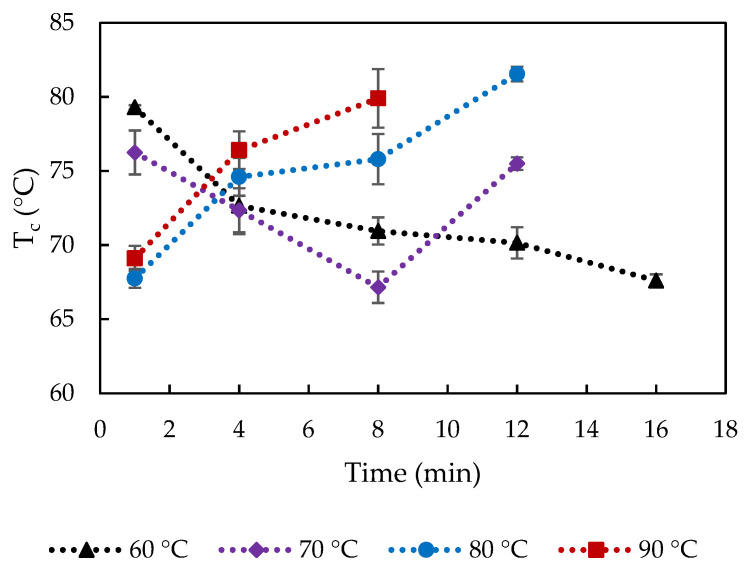
Crossover temperature (T_c_) of curd cooked at varied cooking conditions. The error bars indicate standard deviations.

The crossover temperature of storage and loss moduli, T_c_, (Figure 9) give an indication of the gel-sol transition point of cheese during the temperature sweep test, which can be associated with the point at which the cheese can rapidly melt and flow [28]. The uncooked curd did not show a crossover point (Figure 5), indicating a hard protein matrix structure with small native fat globules that impede melting. All the cooked curds showed values of T_c_ ranging from 67 to 82 °C, which indicates structural modifications caused by the cooking process. It is interesting to note that significantly higher T_c_ was observed after 1 min cooking at 60 and 70 compared to 80–90 °C (*p* < 0.05). Furthermore, T_c_ significantly decreased from 79 to 68 °C during cooking 60 °C, while at cooking at 70 °C, first a decrease in T_c_ from 76 to 67 °C was observed, but suddenly increased after 8 min cooking to 75 °C (*p* < 0.05). During cooking at 80 and 90 °C, an increase of T_c_ is observed along the cooking time from 68 to 82 °C and from 69 to 80 °C, respectively.

These differences were unexpected but might be linked to differences in fat content and entrapped/surface water (from cooking). Higher fat and water content were associated with higher meltability [29,30]. Initially, with cooking at low temperatures 60–70 °C, the T_c_ value likely depended on fat content. Increased % fat content with longer cooking at 60 °C/1–16 min and 70 °C/1–8 min (Figure 1f) could lead to earlier melting, i.e., lower T_c_, and the sudden decrease of fat content from 8 min cooking at 70 °C delayed the crossover of G′ and G′′. Ibáñez et al. (2020) also observed a lower T_c_ in low-fat cheese [25]. As the cooking temperature increased, the rheological properties of curd depend on both water and fat content. The effect of reduced amount of entrapped water in curds cooked at 80–90 °C/1–8 min (Figure 1b) preceded that of increased % fat content on melting, resulting in the higher T_c_ values. The longer holding times might also lead to the formation of new heat-induced calcium-phosphate structures, which might explain the increased T_c_ with extended temperature/time.

### 3.3. Schematic Description of Mass Loss and Structural Changes in Curds as a Consequence of Cooking

**Figure 10:**
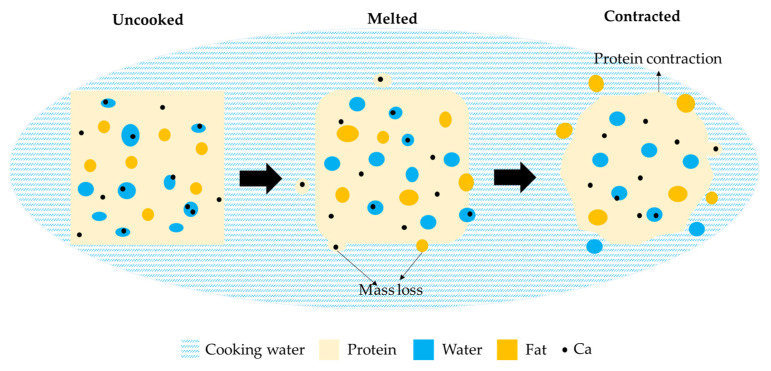
Schematic model proposed for kinetics of mass transfer during cooking of cheese curd.

A schematic model is proposed in Figure 10 to summarize the changes in curd during the water cooking process. The cooking process is complex and depend on: (i) cooking process parameters (e.g., equipment design, curds/water ratio and water agitation); (ii) water properties (e.g., temperature and composition (salt content, Ca, pH)); and (iii) curd properties (size, composition, temperature). 

In this study, three stages were identified to describe the molecular migration and microstructural changes taking place during water cooking. The original curd, consists of a protein (casein) matrix with homogeneously distributed water (serum) and fat globules. Ca is either soluble in the water phase or bound to the protein matrix. When the curd is immersed in water, heat is transferred from the water surrounding the curd cubes to the cube surface, which causes an increase of the water content in the curds. As the surface temperature of the cube increases, the curds surface softens after a few minutes of cooking and the cubes lose the edges due to the water agitation. As heat is transported from the cube surface to the interior of the cube, the curds can then fuse into a continuous curd block once removed from the water. During this stage, protein, fat and Ca are released into the cooking water, but the mass loss seems to occur mainly at surface. Ca becomes more insoluble and is lost together with protein. As cooking proceeds, temperature of the cubes increases, leading to contraction of the protein network due to an increase in hydrophobic interactions and Ca/phosphate-mediated interactions between the para-casein molecules, which causes the observed separated curd appearance. Protein and Ca losses continue at reduced rate compared to the melted stage, whereas the contraction of protein network seems to accelerate fat loss.

In summary, the conditions selected for cooking process can significantly alter cheese curd status and influence its structural and rheological properties. Further studies are needed to further understand changes at molecular and microstructural level.

## 4. Conclusions

Cooking time and temperature induce significant changes in cheese curd composition and rheological properties. Increase of water temperatures from 60 °C to 90 °C and long cooking times significantly accelerate these changes. A significant amount of water coats the curds surface after immersion in water, which has an important role both for mass and heat transfer. The total mass of cooked curds increased up to 10% during the first minutes of cooking, independent of the cooking temperature, although for longer cooking times the mass loss reached 7%. Fat was the main component lost from curd into the cooking water (<3.5% loss based on final curd mass), due to fat liquefaction and migration as the heating intensity increased. Although slight protein and Ca losses were observed, the final concentrations in the cooked curds increased up to 3.4% and 0.07%, respectively. The rate of protein and Ca losses decreased with the cooking time, while the rate of fat loss increased with longer cooking. Thus, fat content in the cooked curds exhibited a sudden decrease after 8 min cooking for all temperatures, which was coincided with the separation phenomenon observed, i.e., inability of curds to fuse. G′ and T_c_ were highly influenced by cooking time and temperature, which were related to loss of water and fat, increase of protein content and increased insoluble Ca-phosphate, and G′ was linearly correlated with increased protein content 21.7–24.9%. 

This cooking study provides initial knowledge about compositional and rheological changes of curd during cooking, and the influence of water temperature and cooking time. Further studies are needed at molecular and microstructural level to further understand the changes observed and to assess the effect of other relevant parameters. 

## Figures and Tables

**Table 1:** Yield (g cooked curd/g initial uncooked curd), reported as means ± standard deviation.

	Yield (G Cooked Curd/G Initial Uncooked Curd)			
Cooking Time (Min)	Water Temperature During Cooking			
	60 °C	70 °C	80 °C	90 °C
1	1.08 ± 0.01 ^Aa^	1.08 ± 0.01 ^Aa^	1.10 ± 0.01 ^Aa^	1.09 ± 0.01 ^Aa^
2	1.05 ± 0.01 ^Ba^	1.07 ± 0.01 ^Aa^	1.06 ± 0.00 ^Ba^	1.08 ± 0.01 ^Aa^
4	1.03 ± 0.01 ^Ca^	1.02 ± 0.01 ^Ba^	1.04 ± 0.01 ^Ba^	1.03 ± 0.02 ^Ba^
8	1.01 ± 0.00 ^Dab^	0.99 ± 0.00 ^Ca^	1.00 ± 0.01 ^Cab^	1.02 ± 0.01 ^Bb^
12	0.98 ± 0.00 ^Ea^	0.94 ± 0.02 ^Da^	0.98 ± 0.02 ^Ca^	0.96 ± 0.01 ^Ba^
16	0.98 ± 0.00 ^Ea^	0.93 ± 0.01 ^Db^	0.93 ± 0.01 ^Db^	0.96 ± 0.01 ^Ba^

^A–E^ Different letters in the same column indicate significantly difference (*p* < 0.05). ^a–b^ Different letters in the same row indicate significantly difference (*p* < 0.05).

**Table 2:** Cooking time (min) when curd started melting, fully melted and separated.

Phase Transition	Water Temperature During Cooking			
	60 °C	70 °C	80 °C	90 °C
**Melting of curds**				
Surface melting	4	2	1	1
Fully fused	-*	4	2	2
**Separation of curds**				
Separated	12	8	8	8

* Curd cooked at 60 °C did not fully fuse.

## Data Availability

Not applicable.
